# Characterization of Surface Modifications in Oxygen Plasma-Treated Teflon AF1600

**DOI:** 10.1021/acs.langmuir.3c03639

**Published:** 2024-02-21

**Authors:** Yijie Xiang, Paul Fulmek, Markus Sauer, Annette Foelske, Ulrich Schmid

**Affiliations:** † Institute of Sensor and Actuator Systems, TU Wien, Gusshausstrasse 27-29, 1040 Vienna, Austria; ‡ Analytical Instrumentation Center, TU Wien, Lehargasse 6, 1060 Vienna, Austria

## Abstract

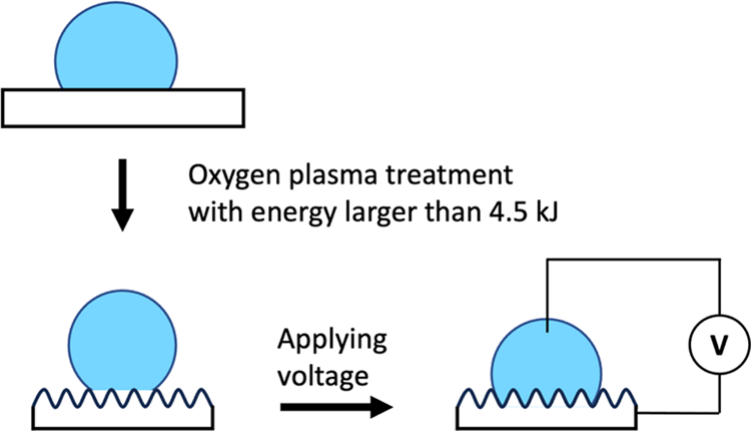

We explore the surface properties of Teflon AF1600 films treated by oxygen plasma with various procedure parameters. Contact angle (CA) measurements, scanning electron microscopy (SEM), atomic force microscopy (AFM), and X-ray photoelectron microscopy (XPS) are employed to investigate the wetting behavior, surface topography, and chemical composition, respectively. While the etched thickness reveals a linear relationship to the applied plasma energy, the surface presents various wetting properties and topographies depending on the plasma energy: low advancing and zero receding CA (1 kJ), super high advancing and zero receding CA (2–3 kJ), and super high advancing and high receding CA (≥4.5 kJ) for the wetting behaviors; pillar-like (≤6 kJ) and fiber-like (>6 kJ) nanoscaled structures for the topographies. The results of XPS analysis reveal slight changes in the presence of O- and F-components (<4%) after oxygen plasma treatment. Furthermore, we discuss the applicability of the Wenzel and Cassie–Baxter equations and employ the Friction-Adsorption (FA) model, where no wetting state and structure-related parameters are needed, to describe the CAs on the plasma-treated surfaces. Additionally, we conduct electrowetting experiments on the treated surfaces and find that the experimental results of the advancing CA are in good agreement with the predictions of the FA model.

## Introduction

Teflon AF1600 is a copolymer comprising tetrafluoroethylene (TFE) and 4,5-difluor-2,2-bis(trifluoromethyl)-1,3-dioxol (PDD) in a ratio of 35:65 mol %. It has exceptional properties, such as high-temperature stability,^1^ excellent chemical resistance and optical characteristics,^2^ along with a low surface energy.^3^ These properties make Teflon AF an ideal candidate for various applications, including electrowetting (EW)-based applications.^4−7^

To modify the surface properties of polymers, such as enhancing wettability for bonding or decreasing wettability for self-cleaning purposes, plasma treatment stands as a widely employed method, alongside other techniques like imprinting/embossing or layer deposition.^8−19^ However, there are few studies related to modifying the surface properties of Teflon AF1600, and exploiting the full potential of this polymer. Sabbatovskii et al. conducted low-pressure argon (Ar) plasma treatment of Teflon AF materials and the results revealed a hydrophilic surface.^20^ Cho et al. investigated the etching rate of Teflon AF by Ar, O_2_, and CF_4_/O_2_ plasma. The resulting surface exhibited an enhanced wettability.^21^

In addition to the limited comprehension of the altered surface properties of Teflon AF, the investigation of the wettability by contact angle (CA) measurements on rough and structured surfaces by plasma treatment presents its own set of challenges. In general, two equations are used for theoretical predictions: the Wenzel equation^22^ and the Cassie–Baxter equation.^23^ The Wenzel equation is used to describe the CA when a droplet completely wets a rough solid surface, while the Cassie–Baxter equation is applied for a solid surface partially wetted by a droplet. However, these equations have specific limitations and uncertainties. One issue is that both equations require parameters derived from the microscopic surface topography, which can be difficult to determine accurately. Additionally, the validity of these equations is debated in the literature, due to various misleading explanation attempts and interpretations. Gao and McCarthy investigated the CAs on partially structured solid surfaces for different triple line locations with respect to the surface structures. The experimental results showed that the CA depends on only the surface structural properties at the triple line. As a consequence, Gao and McCarthy argued that the contact triple line instead of the contact area is important for the determination of CA.^24^ The surface structures presented in their work exhibited inhomogeneity of the surface structures. This inhomogeneity, particularly the difference between the area fraction at the triple line and that across the overall contact area, has sparked discussions regarding the appropriate circumstances for using an area fraction approach.^25−29^ According to Nosonovsky, the Wenzel and Cassie equations are valid only for uniformly rough surfaces,^25^ while McHale suggested that local roughness and local area fractions, instead of global quantities, should be considered.^29^ More discussion regarding the characterization of the CA on rough surfaces can be found in a review paper by Parvate et al.^30^

In this work, we systematically studied the surface properties of Teflon AF1600 after oxygen plasma treatment with different parameters, including treatment time, plasma power, chamber pressure, and gas flow rate, with a focus on the resulting surface topographies and wetting properties. In addition, we applied our previously proposed friction-adsorption (FA) model to describe the CA on these surfaces, where no parameters are required to quantify the structure-specific contact area. Furthermore, we conducted and evaluated the electrowetting (EW) performance of a plasma-treated Teflon AF1600 and verified the applicability of the FA model.

## Experimental Details

### Teflon AF1600 Thin Films

A silicon wafer serves as the substrate for the Teflon AF1600 films. First, a titanium primer is applied by spin coating at 4000 rpm for 1 min using a Süß MicroTech spin-coater, and then annealed at 120 °C for 2 min to enhance the adhesion of the Teflon AF1600 to the silicon substrate.^3^ Next, 4 wt % Teflon AF1600 (DuPont Co.) in fluorinert FC-40 solvent (3 M Company) is spun with 1000 rpm for 60 s and temperature-loaded at 175 °C for 10 min to remove the solvent, subsequently baked at 165 °C for 5 min (glass transition temperature of Teflon AF1600).^3^ The resulting pristine film had a thickness of about 1.4 μm.

### Reactive Ion Etching

A reactive ion etching instrument (STS 320) using a radio frequency power supply at 13.56 MHz is applied in this work to modify the surface properties . The plasma chamber is first evacuated to a pressure of 12 mTorr and purged by nitrogen gas. The treatment process is then conducted by varying the procedure parameters. Next, the plasma chamber is purged again with nitrogen to remove the residual gases for 1 min and pumped down to 12 mTorr for 2 min. A standard treatment process is applied using a treatment time of 30 s, 100 W for plasma power, 20 sccm for oxygen gas flow rate, and 20 mTorr for chamber pressure. To investigate the impact of each parameter on the surface properties, the treatment time, plasma power, oxygen gas flow rate, and chamber pressure are varied from 10 to 120 s, 50 to 400 W, 20 to 30 sccm, and from 20 to 40 mTorr, respectively.

### Surface Properties Characterization

The thickness of the thin films was determined by measuring the step height using a surface profilometer (Dektak).^31^ This involved selectively removing portions of the film to create distinct steps. The surface topography is assessed by scanning electron microscopy (SEM, Hitachi SU8030). The accelerating voltage used in this work ranges from 4 to 8 kV. The beam current is set as 1 μA. A gold layer with a thickness of 10 nm is thermally deposited on top of the surface to minimize charging effects.^11,32,33^ Along with SEM, atomic force microscopy (Bruker Dimension Edge, cantilever: NCHV-A) is employed to characterize the surface topography. In addition, AFM images are taken for the analysis of root-mean-square roughness (*R*_RMS_) and area ratio of the actual surface to the projected surface by Gwyddion.^34^

To comprehensively understand the surface wettability of plasma-treated surfaces, we employed the sessile drop method^35,36^ to measure the advancing and receding CAs using a drop shape analyzer (DSA, Krüss DSA30S). The DSA offers a resolution of 0.01° and an accuracy of 0.1°. A deionized water droplet with a resistivity of 16–18 Mω·cm and an initial droplet volume between 8 and 10 μL, is deposited on the surface. The advancing CA is measured by increasing the droplet volume through the dosing needle at a speed of 0.1 μL/s, while the receding CA is obtained by the droplet evaporation process.^3,37,38^ The CA measurement is also conducted at different temperatures. The detailed setup can be reviewed in our previous work.^3^ In addition to the advancing and receding CA, EW is performed on plasma-treated surfaces, and the response CAs are also measured. More details can be found in the authors’ previous publication.^4^ As the triple line moves during CA measurements, the capillary number, *Ca*, is observed to evaluate the influence of dynamic effects.^35^*Ca*, is calculated by (μ·*v*)/γ,^39^ where μ, γ, and *v* are dynamic viscosity, liquid surface tension, and triple line velocity, respectively. The maximum value of the capillary number in this work is 7 × 10^–6^, thus being considerably smaller than the critical capillary number, 10^–5^. Therefore, dynamic effects during CA measurement are negligible,^35^ and we obtain quasi-static CAs.

X-ray Photoelectron Spectroscopy (XPS) is applied to investigate surface chemical changes after oxygen plasma treatment. The XPS measurements are carried out on a PHI Versa Probe III-spectrometer equipped with a monochromatic Al–Kα X-ray source and a hemispherical analyzer (acceptance angle: ± 22°, angle between the X-ray beam and analyzer: 45°). A combination of automatic electronic and ionic charge compensation was used. Pass energies of 140 and 27 eV and step widths of 0.5 and 0.05 eV are used for survey and detail spectra, respectively. The excitation energy is 1486.6 eV and the beam power and diameter are 25 W and 100 μm, respectively. Data analysis was performed using Multipak software (9.9.1), employing transmission corrections, Shirley backgrounds, and sensitivity factors provided by PHI. Deconvolution of spectra was carried out by using Voigtian line shapes. XPS spectra, including C 1s, O 1s, and F 1s were recorded by running multiple cycles of this sequence, where the average recording time for C 1s is around 12 min.

## Results and Discussion

### Material Removal

**Figure 1:**
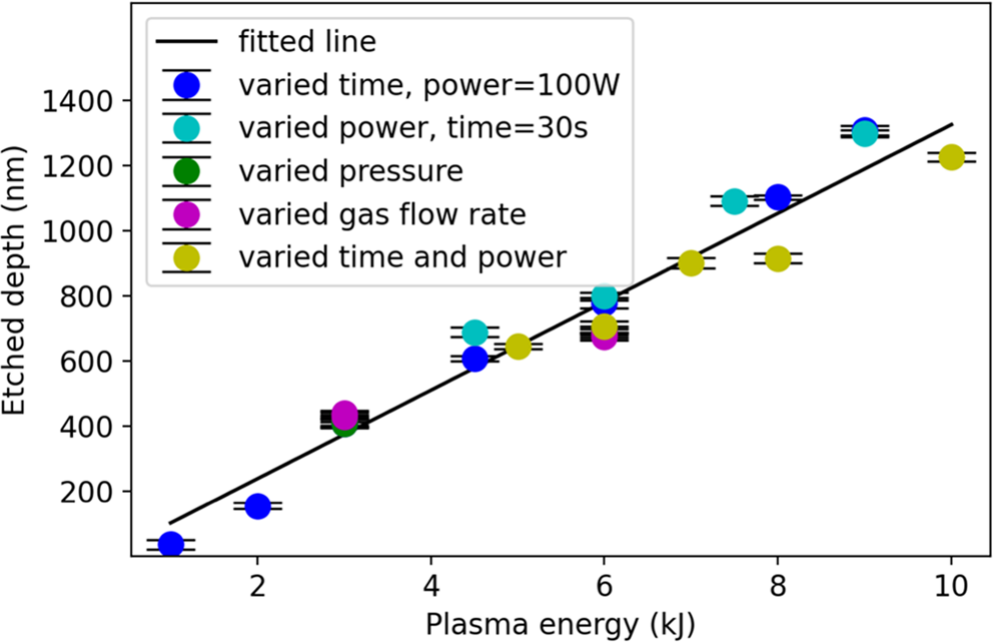
Etched depth of Teflon AF1600 as a function of plasma energy (power ·time). Blue points: plasma power 100 W, pressure 20 mTorr, gas flow rate 20 sccm, treatment time ranging from 10 to 90 s; cyan points: 30 s, 20 mTorr, 20 sccm, power ranging from 100 to 300 W; green points: 30 or 60 s, 100 W, 20 sccm, pressure varies from 20 to 40 mTorr; violet points: 30 or 60 s, 100 W, 20 mTorr, gas flow rate varies from 20 to 30 sccm; olive points: 20 sccm, 20 mTorr, time varies from 20 to 140 s and the power varies from 50 to 400 W.

Within the measurement accuracy, the oxygen gas flow rate and chamber pressure do not affect the etched depth of the Teflon AF1600. The plasma power and treatment time, however, have a significant impact. As illustrated in Figure 1, the etched depth is proportional to the applied plasma energy, which is the product of power and time (in kJ).

### Topography Analysis and RMS Roughness

**Figure 2:**
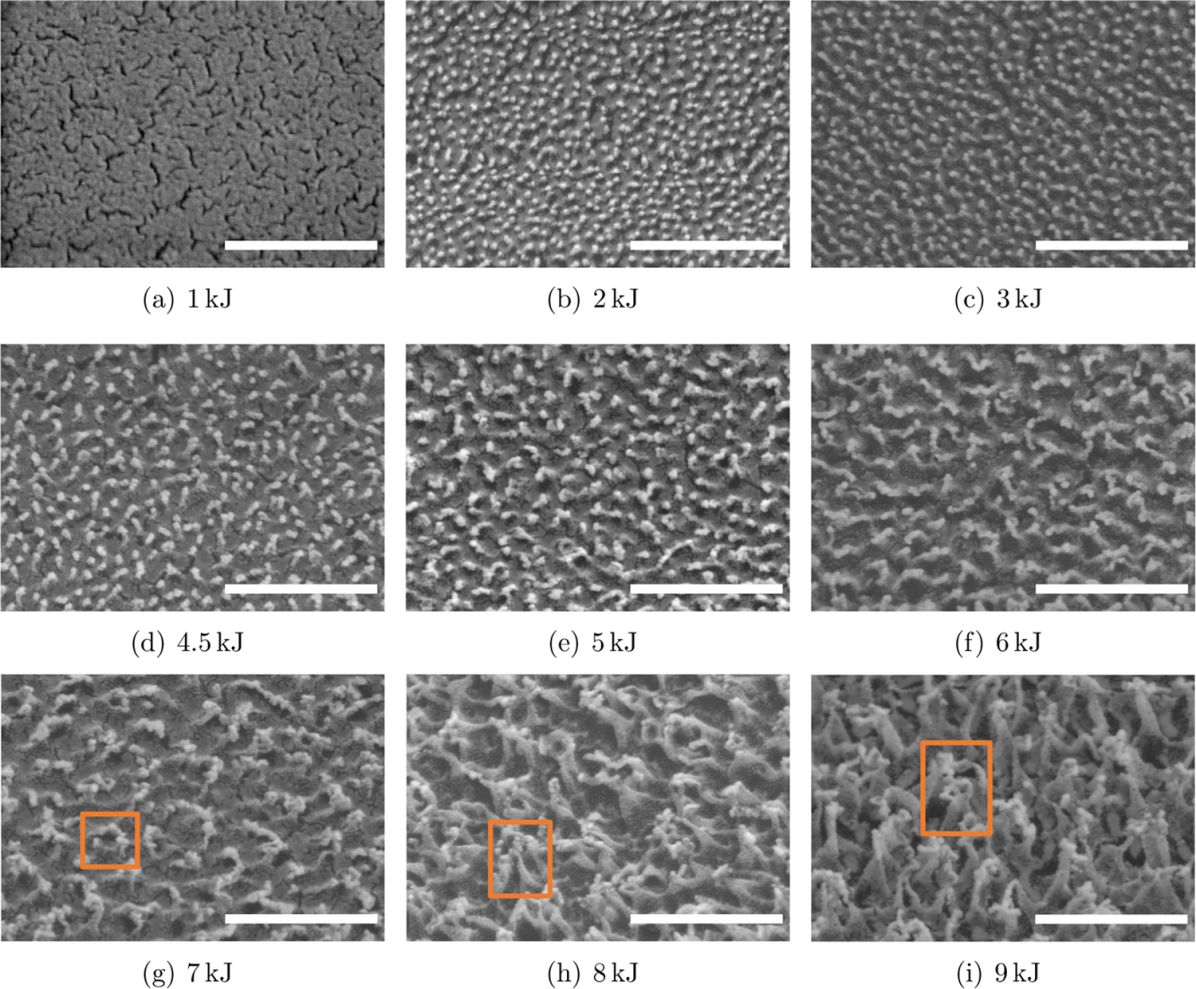
SEM images of Teflon AF1600 surfaces treated by oxygen plasma with energies of (a) 1, (b) 2, (c) 3, (d) 4.5, (e) 5, (f) 6, (g) 7, (h) 8, and (i) 9 kJ. All images are taken by tilting the samples by 30°. The length of the white bar in each image represents 500 nm. The framed area indicates the presence of nanosized structures with undercuts.

**Figure 3:**
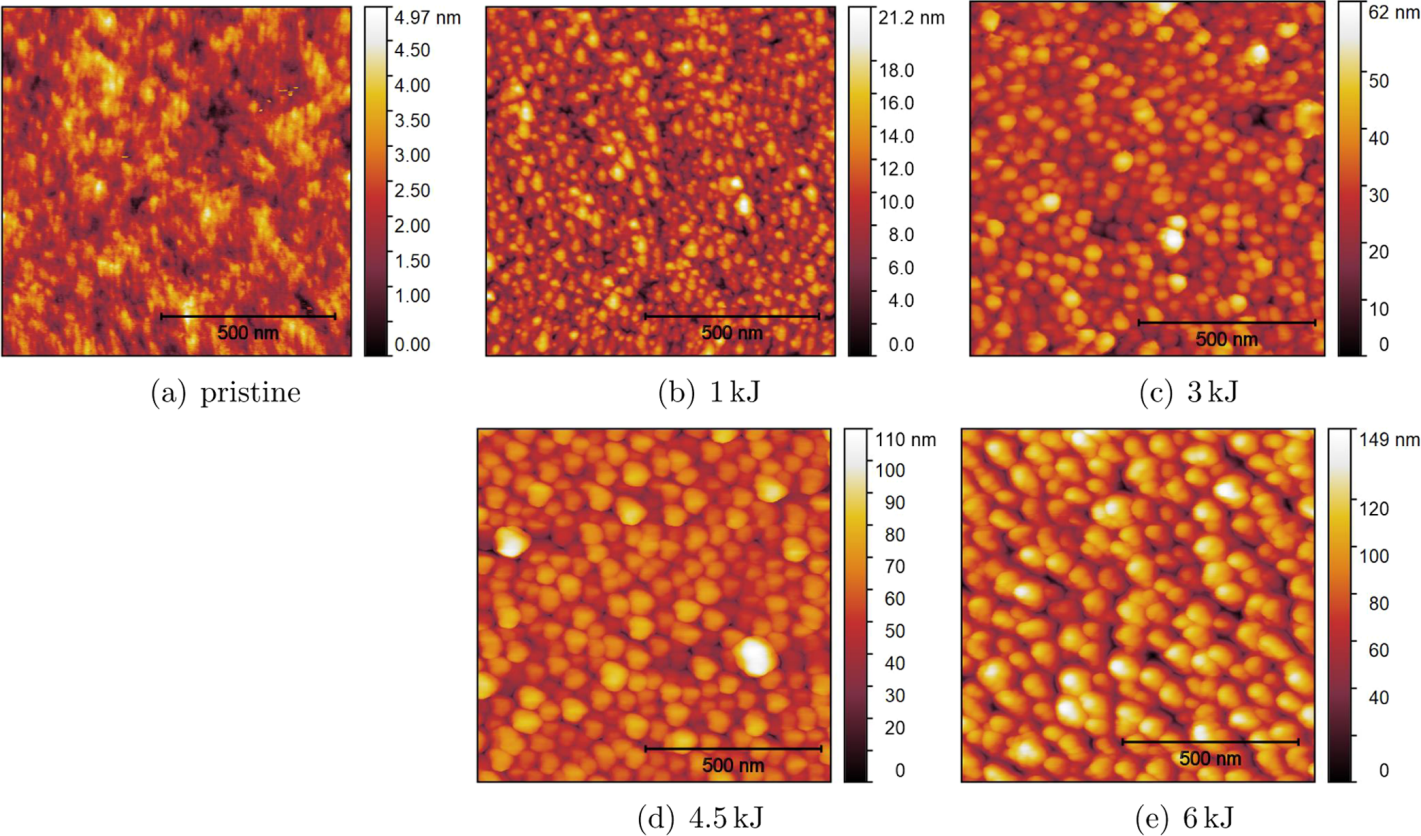
AFM images of (a) untreated surface and plasma-treated Teflon AF1600 surface with plasma energy of (b) 1, (c) 3, (d) 4.5, and (e) 6 kJ.

The surface topography is mainly determined by the plasma energy and remains unchanged with the gas flow rate and chamber pressure. Typical SEM images for surfaces treated by different plasma energies are shown in Figure 2. The results from AFM analyses are illustrated in Figure 3. These results reveal that the oxygen plasma treatment significantly modified the surface and induced nanoscale features depending on the plasma energy. These features are mainly pillar-like and fiber-like structures. For the surface treated with a plasma energy of 1 kJ, the induced surface structures are too small to be clearly observed by SEM. However, AFM measurements reveal the formation of pillar-like structures on the surface, as shown in Figure 3b. When the plasma energy rises to 6 kJ, the pillar-like structures increase both in dimension and height. This trend is evident from the SEM images presented in Figure 2b–f and the AFM images shown in Figure 3c–e. Above this plasma energy, the nanoscaled pillar-like structures further increase in height and are connected on the top while the bottom stays separately. As a result, the pillar-like structures transitioned to fiber-like, and undercuts were created. Figure 2g–i present the surfaces with the fiber-like structures. The framed areas show the undercuts. The presence of the undercuts makes the structures difficult to measure by AFM. Therefore, AFM images were obtained only from samples that are plasma-treated with energies up to 6 kJ, as shown in Figure 3.

**Figure 4:**
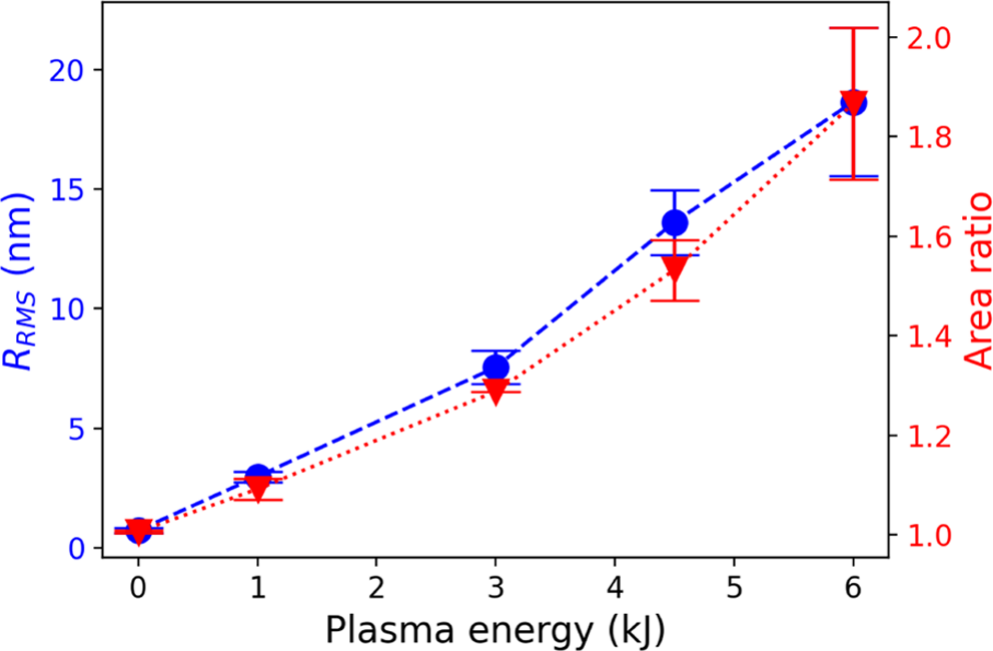
RMS roughness and area ratio of the actual surface to the projected surface as a function of plasma energy. Data are obtained by analysis of AFM images with a scan area of 1 × 1 *μm* by Gwyddion.

The results of the root-mean-square roughness (*R*_RMS_) and area ratio are presented in Figure 4. The results reveal a significant increase in *R*_RMS_, namely, from 0.7 ± 0.09 nm for the pristine surface to 18.7 ± 3.12 nm for the surface treated with a plasma energy of 6 kJ. In addition, the area ratio, which represents the ratio of actual surface area to the projected surface area, also increases significantly with the plasma energy. The area ratio on the pristine surface is 1.006 ± 0.002, while for the surface treated with 6 kJ, it rises correspondingly to 1.867 ± 0.153.

### CA Measurement

**Figure 5:**
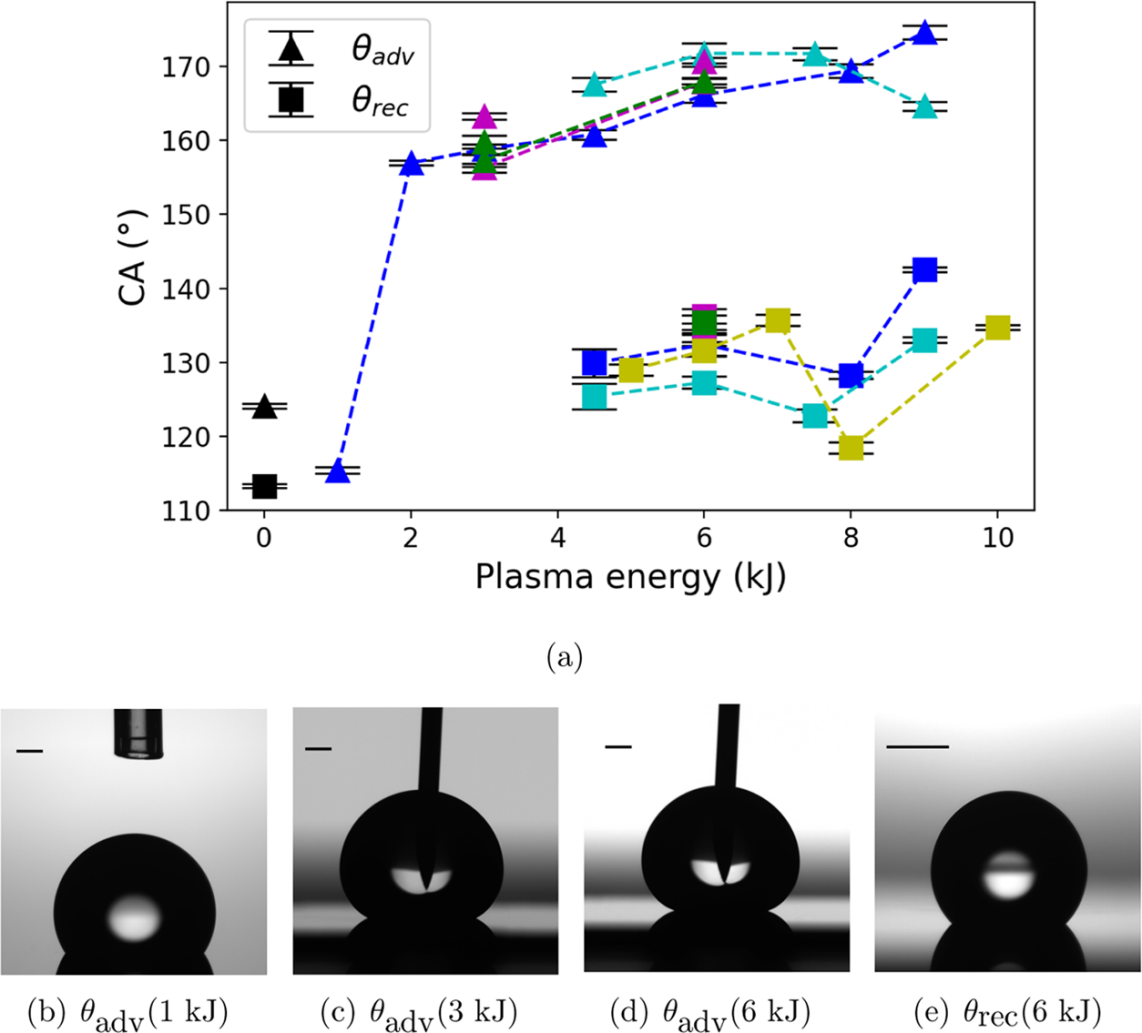
(a) The advancing (triangle points) and receding CA (rectangular points) as a function of the plasma energy at *T* = 25 °C. The points in black represent the θ_adv_ and θ_rec_ on pristine Teflon AF1600.^3^ Points in other colors represent CAs on the oxygen plasma-treated surface with different treatment parameters, and the color coding is identical to that shown in Figure 1. (b) Representative image of water droplets on surfaces treated with a plasma energy of 1 kJ: θ_adv_ = 114.7°; (c) plasma energy of 3 kJ: θ_adv_ = 159.2°; (d) plasma energy of 6 kJ: θ_adv_ = 170.2°; (e) plasma energy of 6 kJ: θ_rec_ = 132.5°. The black bar represents 1 mm. Part of the data (on pristine surfaces) are reproduced from [Temperature Dependence of Water Contact Angle on Teflon AF1600]. Copyright [2022] American Chemical Society.

The wettability of the applied plasma-treated surfaces is primarily determined by the plasma energy. The results of wettability, including advancing and receding CA, are presented in Figure 5. The results show that the plasma-treated surfaces exhibit three different wetting behaviors: (1) low advancing and zero receding CA, (2) super high advancing but zero receding CA, and (3) super high advancing and high receding CA. Here, super high refers to a CA larger than 150°, while high and low refer to values higher and lower than the CAs observed on pristine surfaces.

When the applied plasma energy is low (<1 kJ), the advancing CA (115.5 ± 0.45°) is decreased by oxygen plasma treatment in comparison to the pristine surface (124.1 ± 0.35°). The observed receding CA is 0°, indicating that the triple line sticks to the solid surface and the solid–liquid interface area stays unchanged while the CA decreases continuously. This wetting behavior is referred to as low advancing and zero receding CA. When the plasma energy increases to the range of 2 to 3 kJ, the advancing CAs substantially increase to 157.0 ± 0.31 and 156.3 ± 0.60–163.3 ± 0.46°, while a receding CA of 0° is observed on these surfaces. We refer to these surfaces as having super high advancing but zero receding CA. When the plasma energy further increases, the advancing CA slightly increases (θ > 160°), and a receding CA in the range between 118.5 ± 0.74 and 142.6 ± 0.32° is measured, which is higher than that on the pristine surface (113.3 ± 0.23°). We refer to this wetting behavior as super high advancing and high receding CA.

The Cassie–Baxter and Wenzel equations are commonly used to describe the CA on structured and roughened surfaces, which represent partial and complete contact between the droplet and the surface. The CAs in the Cassie and Wenzel states, denoted as θ_c_ and θ_w_, are described by^22,23^
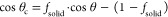
1
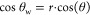
2where *f*_solid_ represents the solid–liquid area fraction across the entire projected area under the droplet, and *r* represents the area ratio of the actual solid area to the projected solid area. θ can be the advancing or receding CA.

In the following discussion, we mainly focus on surfaces that exhibit super high advancing CA and high receding CA. These surfaces are obtained by treating Teflon AF1600 with a plasma energy larger than 4 kJ. The applicability of the Cassie–Baxter and Wenzel equation is evaluated in describing the CAs on these complex surfaces. When the advancing CA is measured, the droplet exhibits a Cassie state. The advancing CA shows a value larger than 160° and is independent of the plasma energy, more specifically, independent of the resulting structure size and accordingly the solid area fraction *f*_solid_. This finding is consistent with previous studies by Kwon et al.^40^ and Öner and McCarthy.^41^ The independency is attributed to the presence of discontinuous structures.^30,40^ The discontinuous structures, such as the pillar-like and fiber-like structures shown in this work, lead to a discontinuous solid–liquid contact, which is separated by solid–air contact interfaces (air pockets). Therefore, a significant energy barrier is presented for the triple line to move over these discontinuous pillars and fibers. As a result, the advancing CA remains high regardless of the solid area fraction and does not follow the Cassie–Baxter equation.

A significant difference of about 30° between the receding and the advancing CAs is observed, as shown in Figure 5, indicating that the droplet-solid interface is in different wetting states. On surfaces with pillar-like structures, the liquid of the droplet can wet from the top along the side walls of the pillar structures to the pocket areas. As a result, a continuous solid–liquid contact is established, indicating the Wenzel wetting state. The receding CA in the Wenzel state can be calculated from eq 2 with the receding CA on the pristine surface (113.3°) and the area ratio, which are obtained from AFM results. The calculated receding CA on surfaces treated by plasma energies with 4.5 and 6 kJ is 127.2 and 137.6°, showing agreement with the experimental findings in Figure 5. It is also important to note that the AFM scanning area is 1 × 1 μm, which is substantially smaller than the solid–liquid contact area with a radius of 1–1.3 mm.

When the structures are more fiber-like (plasma energy >6 kJ), it becomes difficult to obtain precise AFM images as well as an accurate measurement of the area ratio *r*, due to the presence of the undercuts. Consequently, we could not reasonably apply the Wenzel equation to predict the receding CA.

Next, we applied our previously proposed model, the Friction-Adsorption (FA) model,^3,4^ to describe the advancing and receding CA on plasma-treated surfaces. The FA model comprises a temperature-independent friction force *F*_f_ with a limit of *F*_fmax_, and a temperature-dependent water adsorption contribution *F*_ad_, in addition to the three interface tensions for the CA prediction. A proper application of the FA model is based on the assumption that the oxygen plasma treatment predominantly induces topography modification so that the friction force limit *F*_fmax_ is enhanced, while the adsorption contribution *F*_ad_ stays unchanged. A similar approach is suggested by Vandencasteele et al., who conducted XPS experiments on oxygen plasma-treated PTFE. The results revealed that oxygen plasma treatment did not graft new species on the surface of PTFE and the hydrophobicity is therefore enhanced by the increased roughness.^43^ To validate our assumption, we have performed XPS experiments on some selected samples: pristine surface, samples treated with energy of 1, 2, and 6 kJ, which represents the different wetting behaviors: original wetting behavior, low advancing and zero receding CA, super high advancing and zero receding CA, and super high advancing and high receding CA, respectively.

### Chemical Analysis

**Figure 6:**
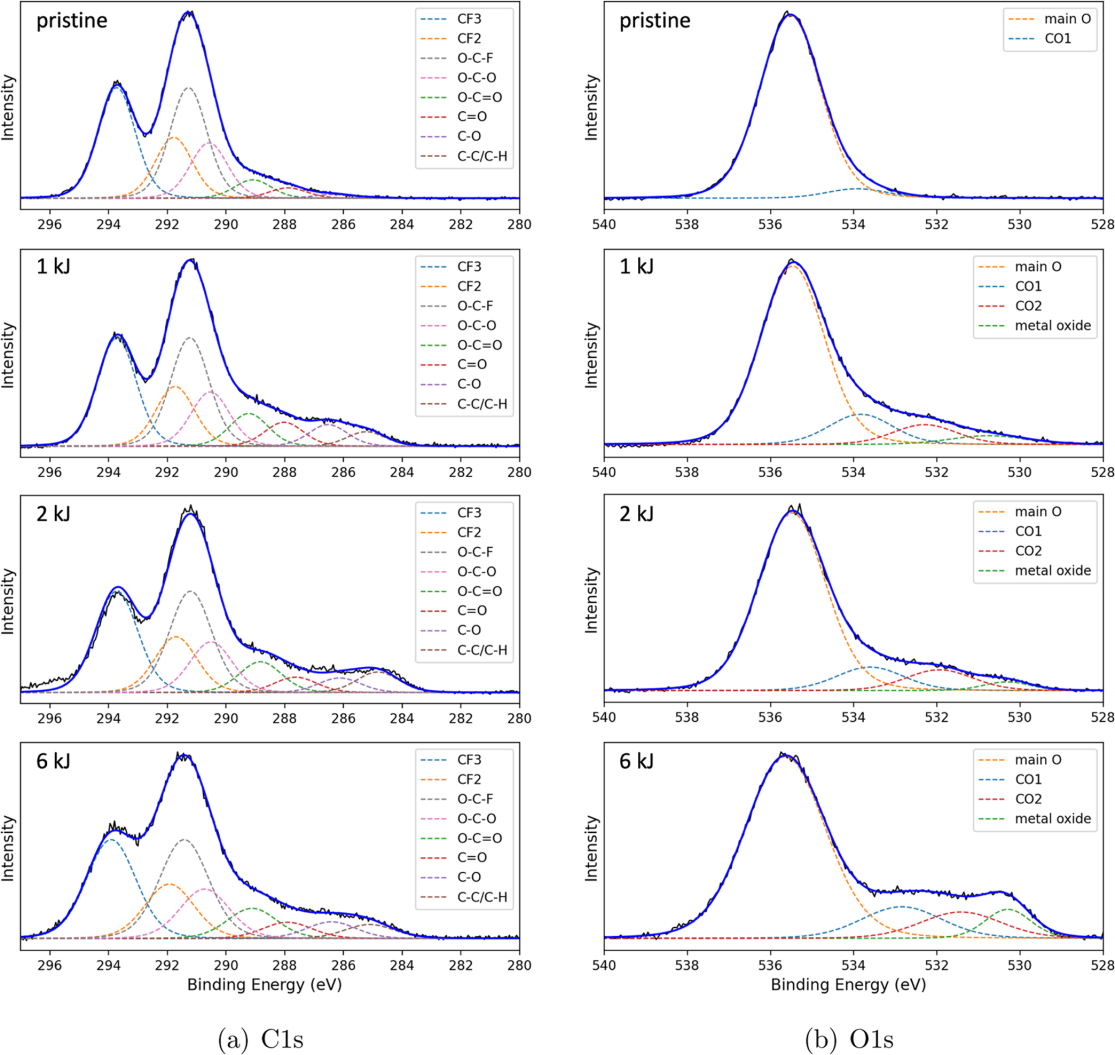
(a) C 1s, (b) O 1s XPS. From top to bottom: pristine Teflon AF1600, and plasma-treated surface by oxygen plasma energy of 1, 2, and 6 kJ, respectively.

**Figure 7:**
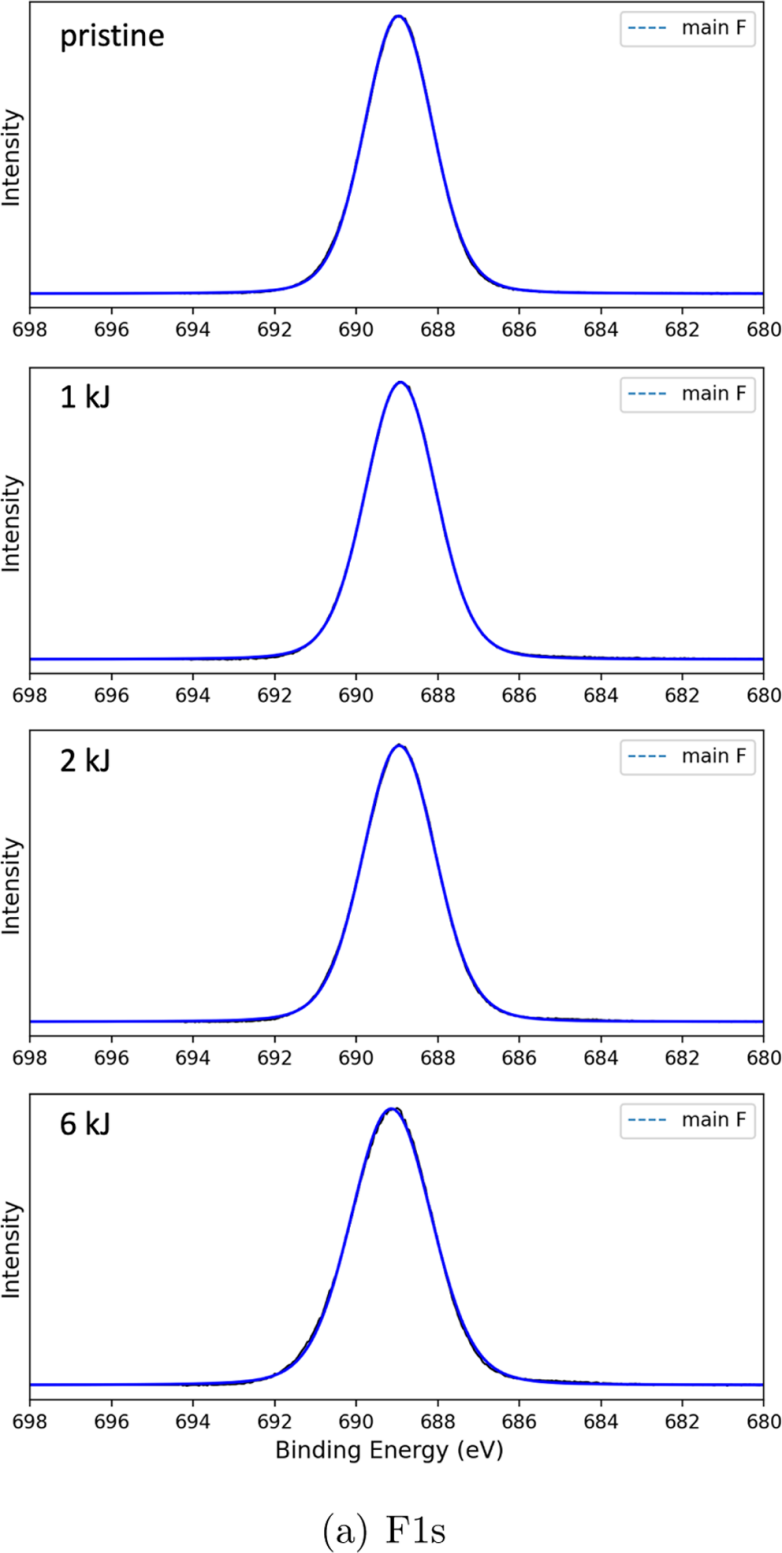
F 1s XPS. From top to bottom: pristine Teflon AF1600, and plasma-treated surface by oxygen plasma energy of 1, 2, and 6 kJ, respectively.

**Figure 8:**
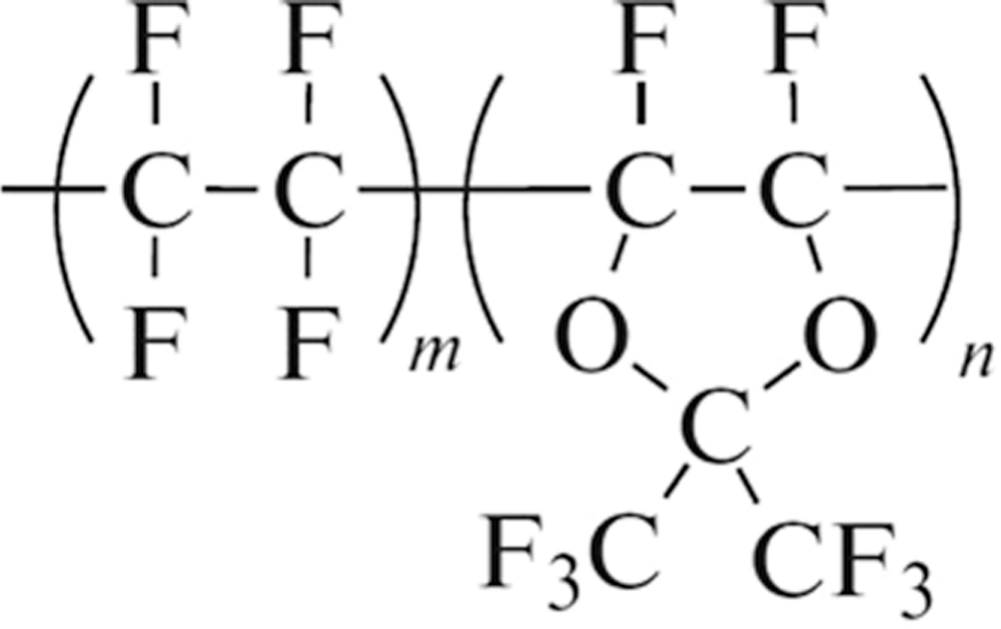
Chemical formula of Teflon AF1600. m, n are mole mass fractions of TFE (65% mol) and PDD (35% mol), respectively.

The results of the XPS investigation are shown in Figures 6 and 7. The C 1s spectra of the pristine Teflon AF1600 surface, as shown in Figure 6a at the top, are characterized by four distinct chemical states of the carbon atoms: CF_3_, CF_2_, and O–C–F, and the O–C–O at 293.8, 291.8, 291.3, and 290.6 eV, respectively. The results show the expected intensity ratios of 2:1:2:1 for CF_3_:CF_2_:O–C–F:O–C–O, based on the chemical formula of Teflon AF1600 shown in Figure 8. In addition, the peaks at 289.1 and 287.9 eV correspond to C–O=O and C=O,^42,44^ indicating surface contamination and/or deterioration of the polymer from exposure to X-rays.^1,43^ After oxygen plasma treatment, the components of Teflon AF1600 were still present in the above-mentioned ratio, while an increase in the amount of oxygen was observed. At 286.4 and 285.1 eV, C–O and C–C/C-H are characterized.^43^ This increase can be attributed to an increase in surface modification resulting from increasing plasma energy.

Figure 6b shows the O 1s spectra. Two components labeled CO1 and CO2, are assigned to CO groups (C=O, C–O, C–=O), which are similar to the deconvolution of the C 1s signal. Moreover, a small amount of titanium oxide is detected, which stems from the Ti primer used during the film spin-coating process (see Experimental Section).

The F 1s spectra, shown in Figure 7, do not show any significant differences between the pristine and plasma-treated samples, indicating the unchanged presence of the functional groups of Teflon AF1600 (Figure 8). This finding is consistent with the observation that the ratio of CF_2_/O–C–O/O–C–F to CF_3_ in the C 1s spectra remains constant.

The results of the quantification analysis are shown in Table 1, revealing a slight decrease in the F and parallel a slight increase in the O concentration (less than 4%). A similar XPS result of PTFE treated by oxygen plasma treatment is revealed by Vandencasteele and Reniers, showing a slight increase of O (less than 5%).^43^

**Table 1:** Surface Composition of Pristine as well as Plasma-Treated Teflon AF1600. T1, T2, and T6 Represent the Sample Treated with an Oxygen Plasma Energy of 1, 2, and 6 kJ

sample	atom %C	atom %F	atom %O
pristine	41.1	48.3	10.6
T1	41.5	46.1	12.4
T2	41.4	46.1	12.5
T6	41.2	44.0	13.9

In the previous discussion, we have shown that the receding CAs on the plasma-treated surfaces with energies of 4.5 and 6 kJ are in agreement with the Wenzel equation. This applicability indicates that the surface has very similar functional groups after oxygen plasma treatment, so that the receding CA on the plasma-treated surfaces can be calculated based on the receding CA on the pristine surfaces (see Wenzel equation, eq 2). Alongside the XPS experimental results, it is reasonable to assume that the oxygen plasma treatment with energy of higher than 4.5 kJ has a limited impact on the chemical compositions and predominatly modifies the topographies of the Teflon AF1600 surfaces.

However, it is important to note that the XPS results are not able to explain the enhanced hydrophilicity in the first and second regimes, where low advancing and zero receding CA, and super high advancing but zero receding CA are exhibited. In the following, the wetting behavior in the third regime of super high advancing and high receding CA is discussed.

### Friction-Adsorption Model

According to the FA model, the advancing and receding CA are written as^3,4^
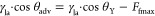
3and
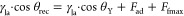
4where the γ_la_ is the liquid–air interface tension, *F*_fmax_ is the friction force limit, and θ_Y_ is the Young’s CA. The *F*_fmax_ on the pristine surface is experimentally determined by previous papers, as *F*_fmax_ = 3.15 ± 0.19 mN/m.^3,4^ θ_Y_ is described by the force equilibrium at the triple line by the three interface tensions.^45^

Combining the advancing and receding CA eqs (eqs 3 and 4), we get
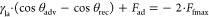
5It is observed that the *F*_fmax_ is independent of temperature, while all other parameters are temperature-dependent.^3,4^ Among these parameters, the liquid–air interface tension, γ_la_(*T*), is well-known^46^ and verified by some of our own experiments. θ_adv_(*T*) and θ_rec_(*T*) were experimentally determined. The temperature-dependent *F*_ad_(*T*) is assumed to be identical to that of pristine Teflon AF1600, as^3,4^
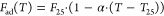
6with *F*_25_ = 4.95 mN/m, α = 0.0182(1/K), and *T*_25_ = 25 °C. Details can be found in the authors’ previous publications.^4^

**Figure 9:**
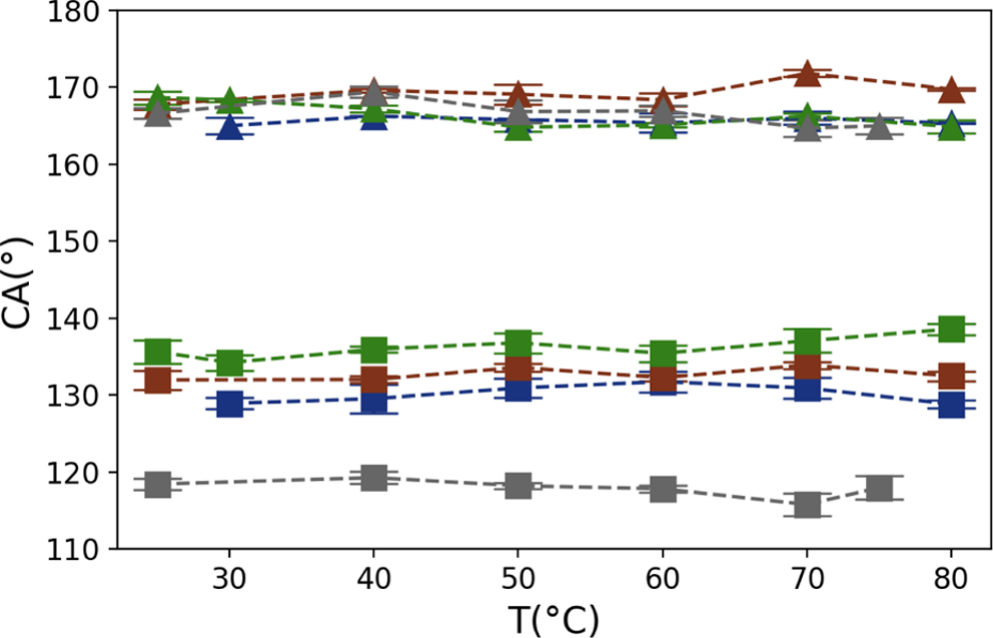
Advancing (triangular points) and receding CAs (rectangular points) of Teflon AF1600 surfaces treated with plasma energies between 5 and 8 kJ as a function of temperature. (blue: 5 kJ, red 6 kJ, green 7 kJ, gray: 8 kJ). The dashed lines are guides for the eyes.

**Figure 10:**
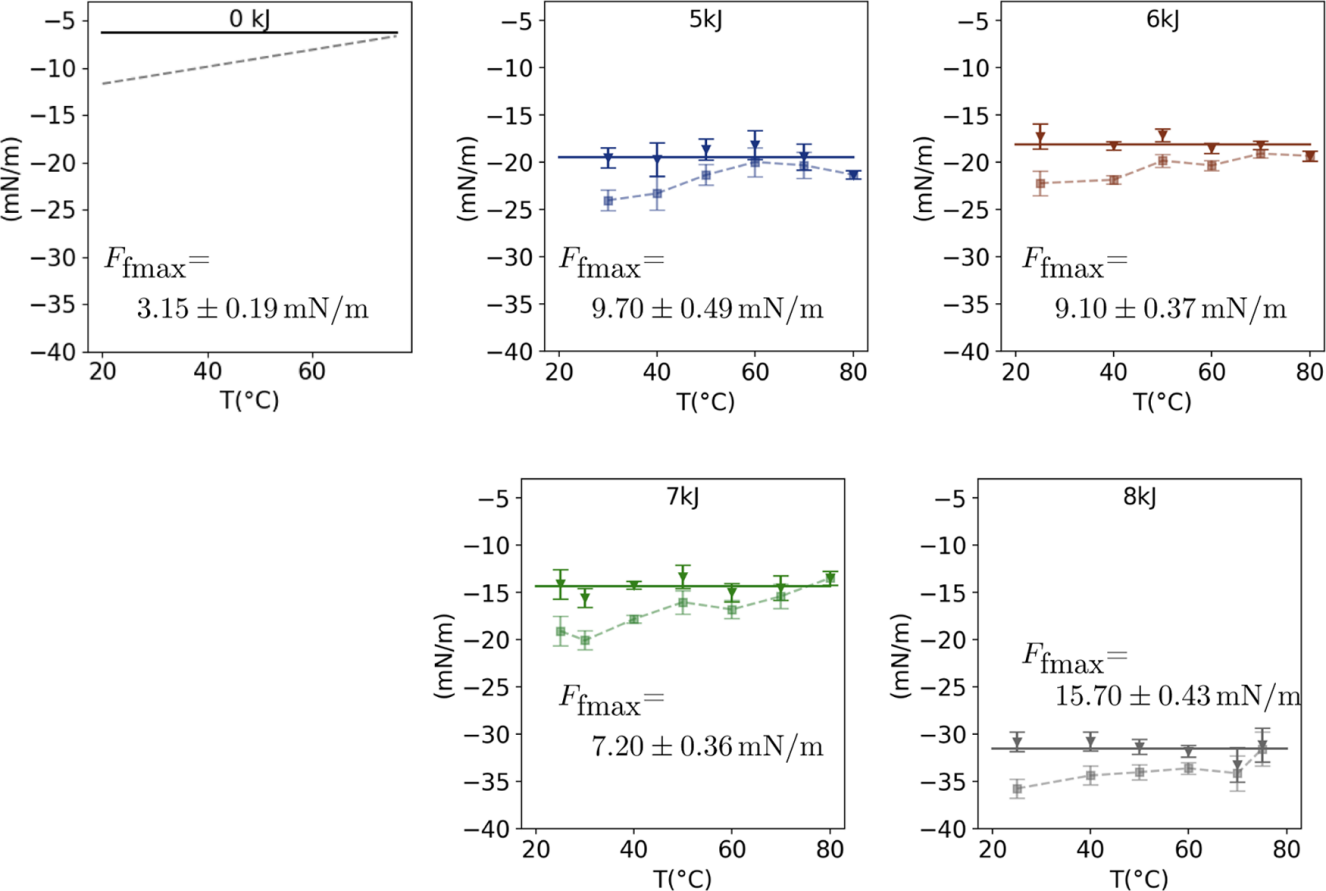
Measurement results of γ_la_·(cos θ_adv_ – cos θ_rec_) (dashed line) and calculated −2·*F*_fmax_ (solid line) on surfaces treated with different oxygen plasma energies as a function of temperature. Part of the data (on pristine surfaces, 0 kJ) are reproduced from [Temperature Dependence of Water Contact Angle on Teflon AF1600]. Copyright [2022] American Chemical Society.

In order to determine the temperature-independent *F*_fmax_, we performed CA measurements at different temperatures on representative plasma-treated surfaces, which exhibit super high advancing and high receding CAs. The results of θ_adv_(*T*) and θ_rec_(*T*) are shown in Figure 9, indicating a constant trend with temperature in the investigated range of 25 to 80 °C. With the known γ_la_, *F*_ad_, and (cos θ_adv_ – cos θ_rec_), the *F*_fmax_ is calculated based on the eq 5. Figure 10 depicts the results of γ_la_·(cos θ_adv_ – cos θ_rec_) (dashed line), as well as the calculated value of γ_la_·(cos θ_adv_ – cos θ_rec_) + *F*_ad_, which is theoretically equal to −2·*F*_fmax_ (solid line). The results distinctly indicate a significant enhancement in the friction force limit (*F*_fmax_) following plasma treatment. For nanoscaled pillar-like structure presented surfaces, *F*_fmax_ maintains a consistent trend with a slight variation: 9.7 ± 0.49 and 9.1 ± 0.37 mN/m for surfaces treated with 5 and 6 kJ. However, the transformation of the resulting structures from pillar-like to fiber-like, achieved by plasma treatment with an energy of 7 kJ, leads to a reduction in *F*_fmax_ to 7.2 ± 0.36 mN/m. A further increase in plasma energy to 8 kJ results in a remarkable rise in the friction force limit (*F*_fmax_) to 15.7 ± 0.43 mN/m, believed to be due to the more predominant pinning sites.

### Electrowetting on Plasma-Treated Surfaces

EW experiments are performed on our Teflon AF layers, as described in detail in a previous paper.^3^ Starting with zero voltage, the CA θ_start_ can be any value between the limits of the advancing and receding CA. With increasing voltage, the CA remains constant, as long as the electrical force *F*_el_ = *c*·*U*^2^/2 is smaller than the friction force limit *F*_fmax_. When *F*_fmax_ is reached, the triple line moves in the advancing direction and the CA follows the advancing CA which is described by the FA model
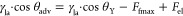
7When decreasing the applied voltage, the CA first stays unchanged due to the existence of the friction force and then follows the receding CA described by
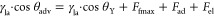
8Details of CA hysteresis during electrowetting can be found in authors’ previous work.^4^

**Figure 11:**
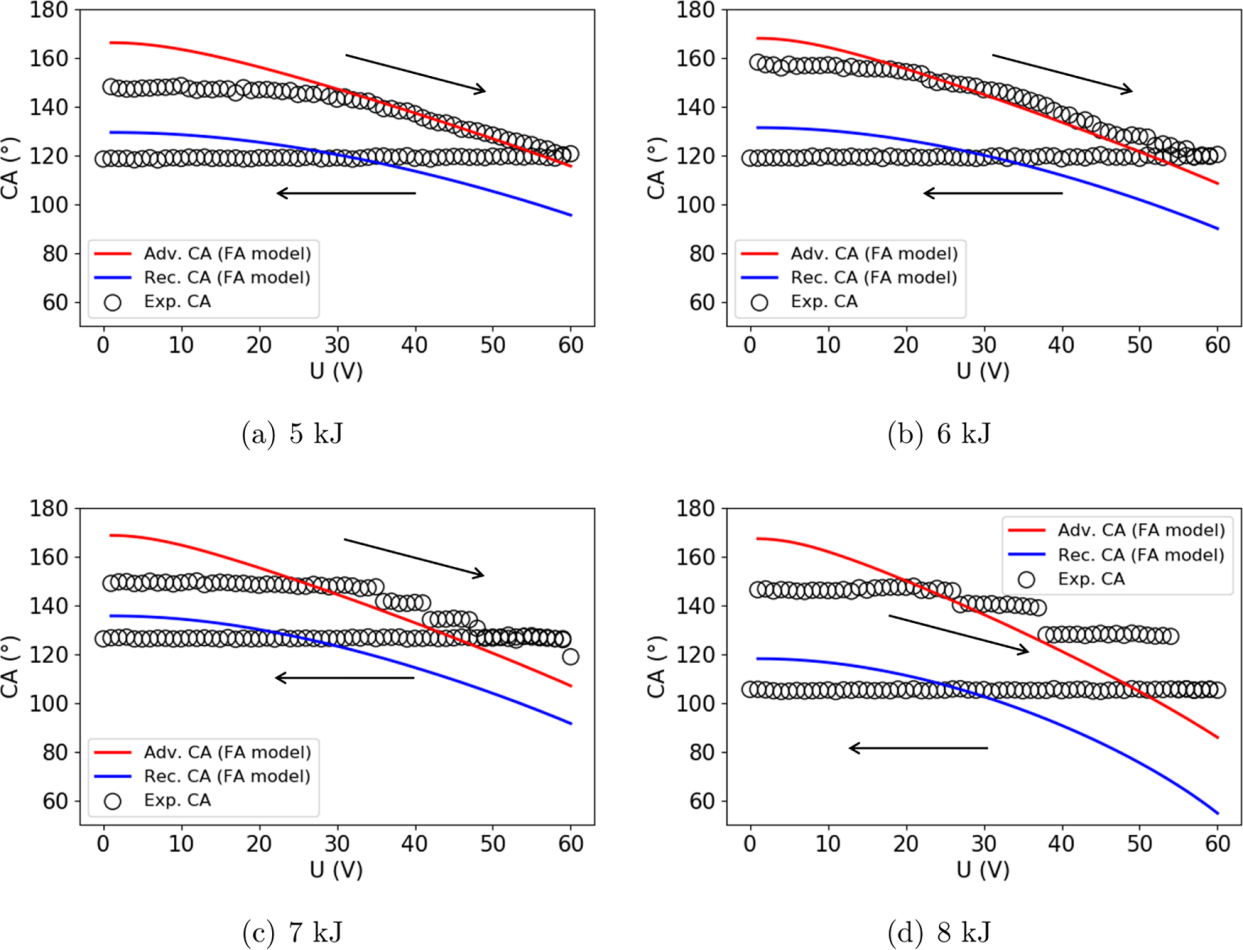
EW response on oxygen plasma-treated surface with the energy of (a) 5, (b) 6, (c) 7, and (d) 8 kJ. The arrows indicate that the applied voltage is increased from 0 to 60 V and then decreased back to 0 V with a voltage change of 1 V/s.

The results of calculated θ_adv_(*U*) and θ_rec_(*U*) are shown in Figure 11 by the red and blue solid lines, respectively, and the experimental results are shown by the black hollow points. When applying an increasing voltage, the CA stays constant first and then follows the advancing CA line. This trend is visible in Figure 11a,b, for the samples which exhibit pillar-like surface structures due to oxygen plasma treatment with energies of 5 and 6 kJ. On surfaces that are treated with higher plasma energies (>6 kJ), the CA is subjected to additional stick–slip phenomena, as shown in Figure 11c,d. The CA stays unchanged when the voltage is increased after reaching the advancing line. This is the so-called stick phase. Then, the CA jumps to another value, which is the so-called slip phase. Although the FA model does not account for the stick–slip phenomena, it successfully predicts the CA after each slipping. This can be observed in Figure 11c,d, where the CAs jump to the advancing CA line following each stick phase. One probable interpretation for the stick–slip phenomena is that the fiber-like structures (see Figure 2) with their undercuts act as pinning sites, leading to the alternated stick and slip phases.^47^

When the applied voltage is decreased, the CA should remain constant until the receding CA line is reached and then follow the receding CA line. In the experimental results (Figure 11), however, this behavior is not visible. The CA remains constant until zero voltage, with a θ_rec_ value smaller than that predicted by the FA model. The reason might be that the nanoscaled structures have a strong trapping effect on ions, and therefore result in an unchanged CA when reducing the voltage.

## Conclusions

In this study, we investigated the surface properties of Teflon AF1600 after oxygen plasma treatment with different parameters. The results demonstrate that the oxygen plasma treatment predominantly leads to linear material removal, directly correlated with the applied plasma energy, and induces the formation of nanoscale features on the surface, while the main chemical functional groups remain unchanged. As a consequence, the CAs on plasma-treated surfaces exhibit various wetting behaviors depending on the plasma energy: low advancing and zero receding CA, super high advancing and zero receding CA, and super high advancing and high receding CA.

The applicability of the Cassie–Baxter and Wenzel equations for describing the CAs on these plasma-treated surfaces is limited. The droplet when measuring the quasi-static advancing CA is in the Cassie–Baxter state, but the θ_adv_ results indicate that the equation is not applicable due to the discontinuous pillar- and fiber-like nanoscaled structures. On the other hand, the Wenzel equation could not be used to predict the receding CA on surfaces with fiber-like structures due to the inability to measure the area ratio resulting from undercuts structures.

We utilized our previously proposed FA model, assuming that the adsorption contribution (*F*_ad_) of the plasma-treated Teflon AF1600 surface is identical with that of the pristine surface. The changed surface topography contributes to the enhancement of the friction force limit (*F*_fmax_). Consequently, the wetting behavior of plasma-treated surfaces can be described by the FA model without any factors related to the structure-area-related parameters (*r* and *f*_solid_) or the wetting states.

Additionally, we validated the accuracy of the FA model through EW experiments. The experimental results of the CAs, obtained by increasing the applied voltage, are in good agreement with the predictions of the FA model. However, the EW-CA stays unchanged when the voltage is decreased from the applied high level. This might be due to the strong trapping effect of the surface structures on the ions.

It is important to note that the FA model is successfully applied to the plasma-treated surfaces, which exhibit super high advancing and high receding CA (energy ≥4.5 kJ). The enhancement of the hydrophilicity in the first and second CA regimes is unclear and could not be explained by XPS or the topography results. Further investigation should be conducted for the first two regimes.

## References

[ref1] Ding S.-J.; Wang P.-F.; Wan X.-G.; Zhang D. W.; Wang J.-T.; Lee W. W. Effects of thermal treatment on porous amorphous fluoropolymer film with a low dielectric constant. Mater. Sci. Eng., B 2001, 83, 130–136. 10.1016/S0921-5107(01)00504-9.

[ref2] Yang M. K.; French R. H.; Tokarsky E. W. Optical properties of Teflon AF amorphous fluoropolymers. J. Micro Nanolithogr. MEMS MOEMS 2008, 7, 033010 10.1117/1.2965541.

[ref3] Xiang Y.; Fulmek P.; Platz D.; Schmid U. Temperature dependence of water contact angle on Teflon AF1600. Langmuir 2022, 38, 1631–1637. 10.1021/acs.langmuir.1c03202. 35048705 PMC8812120

[ref4] Xiang Y.; Fulmek P.; Platz D.; Schmid U. Temperature-dependent electrowetting behavior on Teflon AF1600. J. Mater. Sci. 2022, 57, 15151–15159. 10.1007/s10853-022-07515-y.

[ref5] Wadhai S. M.; Sawane Y. B.; Banpurkar A. G. Electrowetting behaviour of thermostable liquid over wide temperature range. J. Mater. Sci. 2020, 55, 2365–2371. 10.1007/s10853-019-04120-4.

[ref6] Wu H.; Dey R.; Siretanu I.; van den Ende D.; Shui L.; Zhou G.; Mugele F. Electrically controlled localized charge trapping at amorphous fluoropolymer–electrolyte interfaces. Small 2020, 16, 1905726 10.1002/smll.201905726. 31823510

[ref7] Terrab S.; Watson A. M.; Roath C.; Gopinath J. T.; Bright V. M. Adaptive electrowetting lens-prism element. Opt. Express 2015, 23, 25838–25845. 10.1364/OE.23.025838. 26480097

[ref8] Grace J. M.; Gerenser L. J. Plasma treatment of polymers. J. Dispersion Sci. Technol. 2003, 24, 305–341. 10.1081/DIS-120021793.

[ref9] Frøvik N.; Greve M.; Helseth L. Nanostructures and wetting properties controlled by reactive ion etching of fluorinated ethylene propylene. Colloids Surf., A 2019, 574, 228–238. 10.1016/j.colsurfa.2019.04.086.

[ref10] Guo X.; Helseth L. E. Optical and wetting properties of nanostructured fluorinated ethylene propylene changed by mechanical deformation and its application in triboelectric nanogenerators. Mater. Res. Express. 2015, 2, 015302 10.1088/2053-1591/2/1/015302.

[ref11] Powell H. M.; Lannutti J. J. Nanofibrillar surfaces via reactive ion etching. Langmuir 2003, 19, 9071–9078. 10.1021/la0349368.

[ref12] Takahashi T.; Hirano Y.; Takasawa Y.; Gowa T.; Fukutake N.; Oshima A.; Tagawa S.; Washio M. Change in surface morphology of polytetrafluoroethylene by reactive ion etching. Radiat. Phys. Chem. 2011, 80, 253–256. 10.1016/j.radphyschem.2010.07.042.

[ref13] Ryu J.; Kim K.; Park J.; Hwang B. G.; Ko Y.; Kim H.; Han J.; Seo E.; Park Y.; Lee S. J. Nearly perfect durable superhydrophobic surfaces fabricated by a simple one-step plasma treatment. Sci. Rep. 2017, 7, 1981 10.1038/s41598-017-02108-1. 28512304 PMC5434029

[ref14] Xiang Y.; Dejkoski B.; Fulmek P.; Schmid U. Surface properties of μm and sub-μm polydimethylsiloxane thin films after oxygen plasma treatment. Polymer 2023, 275, 125915 10.1016/j.polymer.2023.125915.

[ref15] Carbone E. A.; Boucher N.; Sferrazza M.; Reniers F. How to increase the hydrophobicity of PTFE surfaces using an rf atmospheric-pressure plasma torch. Surf. Interface Anal. 2010, 42, 1014–1018. 10.1002/sia.3384.

[ref16] Wohlfart E.; Fernández-Blázquez J. P.; Arzt E.; del Campo A. Nanofibrillar patterns on PET: the influence of plasma parameters in surface morphology. Plasma Processes Polym. 2011, 8, 876–884. 10.1002/ppap.201000164.

[ref17] Scarratt L. R. J.; Hoatson B. S.; Wood E. S.; Hawkett B. S.; Neto C. Durable superhydrophobic surfaces via spontaneous wrinkling of Teflon AF. ACS Appl. Mater. Interfaces 2016, 8, 6743–6750. 10.1021/acsami.5b12165. 26910574

[ref18] Xiong Z.; Yu H.; Gong X. Designing photothermal superhydrophobic PET fabrics via in situ polymerization and 1, 4-conjugation addition reaction. Langmuir 2022, 38, 8708–8718. 10.1021/acs.langmuir.2c01366. 35776847

[ref19] Miao S.; Xiong Z.; Zhang J.; Wu Y.; Gong X. Polydopamine/SiO2 hybrid structured superamphiphobic fabrics with good photothermal behavior. Langmuir 2022, 38, 9431–9440. 10.1021/acs.langmuir.2c01629. 35875891

[ref20] Sabbatovskii K. G.; Dutschk V.; Nitschke M.; Simon F.; Grundke K. Properties of the Teflon AF1601S surface treated with the low-pressure argon plasma. Colloid J. 2004, 66, 208–215. 10.1023/B:COLL.0000023123.63059.ef.

[ref21] Cho C.-C.; Wallace R.; Files-Sesler L. Patterning and etching of amorphous Teflon films. J. Electron. Mater. 1994, 23, 827–830. 10.1007/BF02651379.

[ref22] Wenzel R. N. Resistance of solid surfaces to wetting by water. Ind. Eng. Chem. 1936, 28, 988–994. 10.1021/ie50320a024.

[ref23] Cassie A. B. D.; Baxter S. Wettability of porous surfaces. Trans. Faraday Soc. 1944, 40, 546–551. 10.1039/tf9444000546.

[ref24] Gao L.; McCarthy T. J. How Wenzel and Cassie were wrong. Langmuir 2007, 23, 3762–3765. 10.1021/la062634a. 17315893

[ref25] Nosonovsky M. On the range of applicability of the Wenzel and Cassie equations. Langmuir 2007, 23, 9919–9920. 10.1021/la701324m. 17665939

[ref26] Panchagnula M. V.; Vedantam S. Comment on how Wenzel and Cassie were wrong by Gao and McCarthy. Langmuir 2007, 23, 13242 10.1021/la7022117. 18001069

[ref27] Gao L.; McCarthy T. J. Reply to “comment on how Wenzel and Cassie were wrong by Gao and McCarthy. Langmuir 2007, 23, 13243 10.1021/la703004v. 18001069

[ref28] Gao L.; McCarthy T. J. Wetting 101°. Langmuir 2009, 25, 14105–14115. 10.1021/la902206c. 19627073

[ref29] McHale G. Cassie and Wenzel: were they really so wrong?. Langmuir 2007, 23, 8200–8205. 10.1021/la7011167. 17580921

[ref30] Parvate S.; Dixit P.; Chattopadhyay S. Superhydrophobic surfaces: insights from theory and experiment. J. Phys. Chem. B 2020, 124, 1323–1360. 10.1021/acs.jpcb.9b08567. 31931574

[ref31] Hafner J.; Benaglia S.; Richheimer F.; Teuschel M.; Maier F. J.; Werner A.; Wood S.; Platz D.; Schneider M.; Hradil K.; et al. Multi-scale characterisation of a ferroelectric polymer reveals the emergence of a morphological phase transition driven by temperature. Nat. Commun. 2021, 12, 152 10.1038/s41467-020-20407-6. 33420070 PMC7794429

[ref32] Gupta N.; Kavya M.; Singh Y. R.; Jyothi J.; Barshilia H. C. Superhydrophobicity on transparent fluorinated ethylene propylene films with nano-protrusion morphology by Ar+ O2 plasma etching: study of the degradation in hydrophobicity after exposure to the environment. J. Appl. Phys. 2013, 114, 164307 10.1063/1.4826897.

[ref33] Herbertson D. L.; Evans C. R.; Shirtcliffe N. J.; McHale G.; Newton M. I. Electrowetting on superhydrophobic SU-8 patterned surfaces. Sens. Actuator A Phys. 2006, 130–131, 189–193. 10.1016/j.sna.2005.12.018.

[ref34] Nečas D.; Klapetek P. Gwyddion: an open-source software for SPM data analysis. Open Phys. 2012, 10, 181–188.

[ref35] Strobel M.; Lyons C. S. An essay on contact angle measurements. Plasma Processes Polym. 2011, 8, 8–13. 10.1002/ppap.201000041.

[ref36] Eral H. B.; t Mannetje D.; Oh J. M. Contact angle hysteresis: a review of fundamentals and applications. Colloid Polym. Sci. 2013, 291, 247–260. 10.1007/s00396-012-2796-6.

[ref37] Bormashenko E.; Bormashenko Y.; Whyman G.; Pogreb R.; Musin A.; Jager R.; Barkay Z. Contact angle hysteresis on polymer substrates established with various experimental techniques, its interpretation, and quantitative characterization. Langmuir 2008, 24, 4020–4025. 10.1021/la703875b. 18302442

[ref38] Bourges-Monnier C.; Shanahan M. Influence of evaporation on contact angle. Langmuir 1995, 11, 2820–2829. 10.1021/la00007a076.

[ref39] Valsamis J.-B.; De Volder M.; Lambert P. Surface Tension in Microsystems; Springer, 2013; pp 3–16.

[ref40] Kwon Y.; Choi S.; Anantharaju N.; Lee J.; Panchagnula M.; Patankar N. Is the Cassie- Baxter formula relevant?. Langmuir 2010, 26, 17528–17531. 10.1021/la102981e. 20964416

[ref41] Öner D.; McCarthy T. J. Ultrahydrophobic surfaces. Effects of topography length scales on wettability. Langmuir 2000, 16, 7777–7782. 10.1021/la000598o.

[ref42] Vandencasteele N.; Merche D.; Reniers F. XPS and contact angle study of N2 and O2 plasma-modified PTFE, PVDF and PVF surfaces. Surf. Interface Anal. 2006, 38, 526–530. 10.1002/sia.2255.

[ref43] Vandencasteele N.; Reniers F. Plasma-modified polymer surfaces: Characterization using XPS. J. Electron Spectrosc. Relat. Phenom. 2010, 178–179, 394–408. 10.1016/j.elspec.2009.12.003.

[ref44] Bolotin I. L.; Tetzler S. H.; Hanley L. XPS and QCM Studies of Hydrocarbon and Fluorocarbon Polymer Films Bombarded by 1- 20 keV C60 Ions. J. Phys. Chem. C 2007, 111, 9953–9960. 10.1021/jp0718000.

[ref45] Young T. III An essay on the cohesion of fluids. Philos. Trans. R. Soc. London 1805, 1, 171–172.

[ref46] Vargaftik N. B.; Volkov B.; Voljak L. International tables of the surface tension of water. J. Phys. Chem. Ref. Data 1983, 12, 817–820. 10.1063/1.555688.

[ref47] Reid R. C.; Merrill M. H.; Thomas J. P. Stick–slip behavior during electrowetting-on-dielectric: polarization and substrate effects. Microfluid. Nanofluid. 2020, 24, 1–9. 10.1007/s10404-020-02374-y.

